# Characterization and trans-generation dynamics of mitogene pool in the silver carp (*Hypophthalmichthys molitrix*)

**DOI:** 10.1093/g3journal/jkae101

**Published:** 2024-06-26

**Authors:** Jinlin Li, Hengshu Wu, Yingna Zhou, Manhong Liu, Yongheng Zhou, Jianing Chu, Elizabeth Kamili, Wenhui Wang, Jincheng Yang, Lijun Lin, Qi Zhang, Shuhui Yang, Yanchun Xu

**Affiliations:** College of Wildlife and Protected Area, Northeast Forestry University, Harbin 150040, China; National Forestry and Grassland Administration Research Center of Engineering Technology for Wildlife Conservation and Utilization, Harbin 150040, China; College of Wildlife and Protected Area, Northeast Forestry University, Harbin 150040, China; National Forestry and Grassland Administration Research Center of Engineering Technology for Wildlife Conservation and Utilization, Harbin 150040, China; College of Wildlife and Protected Area, Northeast Forestry University, Harbin 150040, China; National Forestry and Grassland Administration Research Center of Engineering Technology for Wildlife Conservation and Utilization, Harbin 150040, China; College of Wildlife and Protected Area, Northeast Forestry University, Harbin 150040, China; National Forestry and Grassland Administration Research Center of Engineering Technology for Wildlife Conservation and Utilization, Harbin 150040, China; College of Wildlife and Protected Area, Northeast Forestry University, Harbin 150040, China; National Forestry and Grassland Administration Research Center of Engineering Technology for Wildlife Conservation and Utilization, Harbin 150040, China; College of Wildlife and Protected Area, Northeast Forestry University, Harbin 150040, China; National Forestry and Grassland Administration Research Center of Engineering Technology for Wildlife Conservation and Utilization, Harbin 150040, China; College of Wildlife and Protected Area, Northeast Forestry University, Harbin 150040, China; National Forestry and Grassland Administration Research Center of Engineering Technology for Wildlife Conservation and Utilization, Harbin 150040, China; College of Wildlife and Protected Area, Northeast Forestry University, Harbin 150040, China; National Forestry and Grassland Administration Research Center of Engineering Technology for Wildlife Conservation and Utilization, Harbin 150040, China; College of Wildlife and Protected Area, Northeast Forestry University, Harbin 150040, China; National Forestry and Grassland Administration Research Center of Engineering Technology for Wildlife Conservation and Utilization, Harbin 150040, China; College of Wildlife and Protected Area, Northeast Forestry University, Harbin 150040, China; National Forestry and Grassland Administration Research Center of Engineering Technology for Wildlife Conservation and Utilization, Harbin 150040, China; College of Wildlife and Protected Area, Northeast Forestry University, Harbin 150040, China; National Forestry and Grassland Administration Research Center of Engineering Technology for Wildlife Conservation and Utilization, Harbin 150040, China; College of Wildlife and Protected Area, Northeast Forestry University, Harbin 150040, China; College of Wildlife and Protected Area, Northeast Forestry University, Harbin 150040, China; National Forestry and Grassland Administration Research Center of Engineering Technology for Wildlife Conservation and Utilization, Harbin 150040, China

**Keywords:** silver carp, *Hypophthalmichthys molitrix*, heteroplasmy, mitogene pool, trans-generation dynamics

## Abstract

Multicopied mitogenome are prone to mutation during replication often resulting in heteroplasmy. The derived variants in a cell, organ, or an individual animal constitute a mitogene pool. The individual mitogene pool is initiated by a small fraction of the egg mitogene pool. However, the characteristics and relationship between them has not yet been investigated. This study quantitatively analyzed the heteroplasmy landscape, genetic loads, and selection strength of the mitogene pool of egg and hatchling in the silver carp (*Hypophthalmichthys molitrix*) using high-throughput resequencing. The results showed heteroplasmic sites distribute across the whole mitogenome in both eggs and hatchlings. The dominant substitution was Transversion in eggs and Transition in hatching accounting for 95.23%±2.07% and 85.38%±6.94% of total HP sites, respectively. The total genetic loads were 0.293±0.044 in eggs and 0.228±0.022 in hatchlings (P=0.048). The d*N*/d*S* ratio was 58.03±38.98 for eggs and 9.44±3.93 for hatchlings (P=0.037). These results suggest that the mitogenomes were under strong positive selection in eggs with tolerance to variants with deleterious effects, while the selection was positive but much weaker in hatchlings showing marked quality control. Based on these findings, we proposed a trans-generation dynamics model to explain differential development mode of the two mitogene pool between oocyte maturation and ontogenesis of offspring. This study sheds light on significance of mitogene pool for persistence of populations and subsequent integration in ecological studies and conservation practices.

## Introduction

Mitochondrial genome is a multicopied and maternally inherited extranuclear genetic material with significant metabolic functions such as DNA replication and transcription, RNA processing and translation, and post-translation maturation of newly synthesized polypeptides (Taanman 1999). Notably, there has been increasing interest to explore mechanisms and dimensions related to the existence of multiple mitochondrial DNA haplotypes in a cell, tissue, organ, and individual, technically termed heteroplasmy (Solignac *et al.* 1983; Ujvari *et al.* 2007; Wen *et al.* 2016; Mastrantonio *et al.* 2019; Parakatselaki and Ladoukakis 2021; Stewart and Chinnery 2021). Interrelatedly, the total variants at each level are regarded as a mitogene pool (Wang *et al.* 2023) exhibiting spatial and temporal dynamics across different stages of life history. The development of mitogene pool occurs during two different stages, (1) the egg stage that happens prior to fertilization, and (2) the ontogenic stage which happens during embryonic and postnatal development, by adding *de novo* mutations and amplifying a part of them through replication. The generation of *de novo* mutations is mainly the function of replication-linked transition mutations (Kreutzer and Essigmann 1998; Zheng *et al.* 2006; Sanchez-Contreras *et al.* 2021), reactive oxygen species (ROS)-linked, and subsequent repair-linked mutations (Shokolenko *et al.* 2009; Fontana and Gahlon 2020).

Typically, an egg contains large number of mitogenomes (Otten and Smeets 2015; Read *et al.* 2021) constituting the egg mitogene pool, which is the initial *seeds* of individual mitogenomes if the contribution of paternal mitogenomes is neglected (Hutchison *et al.* 1974; Sato and Sato 2012, 2017). Because all variants may participate in the reaction chains of energy production in the mitochondria, both the volume (number of variants) and quality (beneficial, neutral, and deleterious mutations) of mitogene pool may influence the function of oocyte, somatic cells, and subsequent fitness of individuals (Wang *et al.* 2023). In turn, the cell or organ’s function shapes the volume and quality by means of purifying and/or positive selection to make it compatible for the functional requirements (Zhang *et al.* 2018). During transition from egg to offspring, only a tiny fraction of mitogenomes in the egg can be recruited and inherited by the offspring, forming a severe bottleneck and drift in variants composition in offspring (Li *et al.* 2016; Otten, Theunissen, *et al.* 2016; Schaack *et al.* 2020). Under such circumstances, the egg mitogene pool ought to be different from the offspring’s in volume, quality, mode, and strength of selection. These make the two mitogene pools a novel domain to explorethe descendibility of mitogenomes and its functional consequence in relation to individual fitness (Parakatselaki and Ladoukakis 2021). In fact, the traditional haplotype defined for an individual is the consensus sequence of all variants in its mitogene pool (Wang *et al.* 2023). When we move eyes to the whole mitogene pool and its trans-generation dynamics, we may deepen the insights into ontogenic history, potential fitness, and contemporary response to environmental factors at individual level. This would expand our conception on mitogenomic diversity from traditional focuses on the diversity at population level and retrospection of evolutionary history to a new range and finally incorporate the mitogene pool into ecological studies and conservation practices.

The silver carp (*Hypophthalmichthys molitrix*) is naturally distributed in Asia and currently occurs in almost 90 countries in the world by introduction and invasion (Liu *et al.* 2012; Zhu *et al.* 2023). Each female silver carp can produce a large quantity of small eggs in a breeding season. This phenomenon makes it a convenient model to analyze the mitogene pool of the eggs and offspring from the same female. We comparatively investigated the characteristics, selection mode and strength and genetic load of the mitogene pool of eggs and hatchling, and probed into the trans-generation dynamics and quality control mechanism of mitogene pool of fish.

## Materials and methods

### Collection of silver carp eggs and hatchlings

Six female silver carps aged 10–16 years were randomly chosen from a fishery farm. Ovulation was induced using human chorionic gonadotropin (HCG) according to the procedure in Li *et al.* (2008), and eggs were randomly collected from each female and a portion of them were stored in liquid nitrogen. Artificial fertilization was then performed by a skilled technician on the remaining eggs using fresh semen collected from an adult male. The fertilized eggs were then incubated in tanks with circulating water at 28^∘^C, one tank for a female’s eggs. Three days later, hatchlings were randomly collected from each tank/female and immediately stored in liquid nitrogen. Animal maintenance and experimental procedures were approved by ethics committee at Northeast Forestry University (approval no. 2024049).

### DNA isolation, library construction, and whole genome resequencing

In order to avoid the bias of sequencing due to the small quantity of mtDNA per egg, impacts of yolk on DNA extraction efficiency, and obtain sufficient DNA for library construction, 500 eggs from each female carp were pooled and subjected to DNA isolation using commercial AxyPrep Genomic DNA Mini Kit (Axygen, USA) according to the manufacturer’s instructions.

To eliminate the impact of yolk DNA in the yolk sac that locates below the head and belly of hatchlings, the head and belly of hatchlings were removed using a clean scalpel leaving approximately 2 mm body and tail for DNA extraction. DNA isolation from 100 hatchling pools per female was performed as above.

The extracted DNA from eggs and hatchlings were quantified using Qubit 4.0 (Thermo, USA) and sheared into 250–350 bp fragments in an ultrasonic crusher (Covaris, USA) and subjected to library construction. Library construction was performed using the MGIEasy universal DNA Library Preparation kit (BGI, China) including procedures of end repair, adaptors ligation, and PCR amplification according to the manufacturer’s instructions (MGI Tech Co., Ltd. 2019). The library was sequenced using 100-bp pair-end mode on a DNBSEQ-T1 sequencer (BGI, China).

### Sequencing data preprocessing

The raw data were filtered using Fastp v0.22 (Chen *et al.* 2018) to remove reads containing unknown bases (N) and with quality scores below 20 (Q20). The clean reads of each sample were aligned by bwa mem v0.7.8 (Li and Durbin 2009) to the reference chromosomal genome (NCBI: GCA_022817975.1) and mitogenome (NCBI: NC_010156) to (i) compute the coverage and sequencing depth and (ii) to reserve pure mtDNA reads for further assembly of individual reference mtgenome for variants identification. The reserved pure mtDNA reads of the eggs and hatchlings derived from the same female carp were pooled, and 50% were randomly subsampled using NovoPlasty v4.3.1 software (Dierckxsens *et al.* 2017) for assembly of individual reference mtgenome. Individual reference mtgenome was then assembled, annotated, and corrected using the mitos website (http://mitos2.bioinf.uni-leipzig.de/iNDex.py) (Bernt *et al.* 2013) and the blastn program.

### Variants calling

NovoPlasty is currently the dominant software for detecting mtDNA varients or heteroplasmy, but the minimum detection threshold of 0.6% may miss many rare mtDNA mutations in eggs or hatchlings (Dierckxsens *et al.* 2020; Bi *et al.* 2023), while SAMtools is more sensitive to low-frequency variants (Liu *et al.* 2019). To improve reliability and robustness, we tested the ability of the two assays using one pure mtDNA reads pool of hatchlings. As expected, we found that 96.04% of varients detected by NovoPlasty (total of 101) were contained by SAMtools (total of 166) (Supplementary Fig. S1), which could detect more low-frequency variants (total of 51, minor allele frequency, MAF <0.6%) that were not detected by NovoPlasty (Supplementary Fig. S1). We then found that these low-frequency variants can not be detected using SAMtools in another hatchling pool (4 of total 133 varients MAF <0.6%), meaning that they were not errors introduced by sequencing. Finally, the mpileup program of SAMtools v1.17 (Danecek *et al.* 2021) and the pileup2cns program of VarScan v2.4.4 (Molina-Mora and Solano-Vargas 2021) were used to map the pure mtDNA reads of each sample of eggs and hatchlings to the corresponding individual reference mtgenome for varients calling. Varients were filtered with the criteria base quality value >20, mapping quality value >30, MAF >0.1%, P−value<0.05 and sequencing depth greater than the median depth of the total varients in this sample. Finally, the filtered varients were regarded as heteroplasmic sites (HP sites) and the number and frequency (equal to MAF) of each site were counted for each sample.

### Detection of nuclear mitochondrial DNA segments

The positions and lengths of the nuclear mitochondrial DNA segments (NUMTs) (Tsuji *et al.* 2012) were identified using blastn and lastz software with e-values of 0.001 and 0.0001, respectively, with reference to the method in Antunes and Ramos (2005).

### Computation of selection pressure

SnpEff v5.0.1 (Cingolani 2022) was used to identify nonsynonymous and synonymous substitutions on all mtDNA coding sequence (mtCDS). Then, the selective pressure was identified by the ratio of nonsynonymous substitution rate (dN, sum of nonsynonymous mutation frequency) to synonymous substitution rate (dS, sum of nonsynonymous mutation frequency) (Moradian *et al.* 2007).

### Calculation of the average number of mitogenomes per egg and somatic cell

The silver carps we studied are diploid (2n=48) (Bhatnagar *et al.* 2019), and the eggs are haploid cells with a single copy of chromosomes. Assuming chromosomal and mitochondrial DNA are handled and sequenced with no significant differences, average sequencing coverage should be proportional to DNA copy number for chromosomal and mtDNA (Ding *et al.* 2015). As a proof of principle, we observed the average depth of coverage across the 24 chromosomes for each pool of eggs. As expected, sequencing depth was largely flat across 24 chromosomes (Supplementary Fig. S2). Therefore, the number of mtDNA copies per egg (Ce) was inferred as:


(1) $$C_{e}=\frac{\text{mtDNA average coverage}}{\text{chromosomal DNA average coverage}}.$$

Since the somatic cells of the hatchlings are biploid with two copies of chromosomes, and the average depth of coverage across the 24 chromosomes for each sample is essentially equal (Supplementary Fig. S2), the number of mtDNA copies per somatic cell of the hatchlings (Cf) was inferred as:


(2) $$C_{f}=\frac{2\times (\text{mtDNA average coverage})}{\text{Chromosomal DNA average coverage}}.$$

### Estimation of genetic load on mitogenomes

Mutations leading to loss of protein function (LOF) and impaired but incomplete loss of protein function (deleterious missense mutations) were considered as genetic loads. We calculated the number of LOF mutations based on SnpEff annotation of all HP sites in mtCDS, including stop gained variant, splice acceptor variant and splice donor variant (Hu *et al.* 2020). Subsequently, the missense mutations obtained by SnpEff annotation were scored using the Grantham matrix of R package “grantham”. Those with a score >150 were regarded as deleterious missense mutations and the number was counted (Grantham 1974). The total genetic loads were then obtained by adding the number of LOF and deleterious missense mutations. Finally, by dividing the total genetic loads by the total number of SNPS, we obtained the ratio of genetic load as a measure of the quality of the mitogene pool.

### Statistical analysis

Data were organized using Microsoft Excel 2019 software. A Shapiro–Wilk test model was used to test for data normality, and a Paired Samples t-test model was used to test for differences in mitogene pool volume, quality, and selection pressure between eggs and hatchlings from the same female. Statistical analysis was performed using R v4.3.1 with the significance level of α=0.05 for all tests and comparisons. Graphical presentation was also created using R v4.3.1.

## Results

### Sequencing statistics

All samples were successfully sequenced with a total of 46.17±1.33 Gb raw data for eggs per female fish and 50.50±6.72 Gb raw data for hatchlings per female fish, >90.41% reads above Q30. The sequencing depth of egg’s and hatchlings mitogenomes was 7,789–7,820× and 853–4,565×, respectively, with the coverage of 100% for both.

### NUMTs detection

With the reference of nuclear genome (GCA_022817975.1) and mitogenome (NC_010156) of silver carp, a total of 4 Numt sequences were detected (Table 1). They were then removed from the sequence data from further heteroplasmy analysis.

**Table 1.** Numts detected from the genomes of silver carps.

Software	MtDNA position	NuDNA position	E value	Length (bp)
Blastn	9,631–9,663	1,178,841–1,178,873	0.006	33 bp
Blastn	16,494–16,520	14,423,251–14,423,225	0.006	27 bp
Blastn	16,496–16,529	14,146,527–14,146,495	0.006	34 bp
Lastz	2,122–2,268	950,691–950,836	0.0001	147 bp

### The number of mitogenome per egg and per somatic cell

The five samples showed the average number of mitogenome per egg (Ce) varied between 1,374.8 and 2,414.91 copies, while the average number per cell of hatchlings (Cf) from the same female carp varied from 25.04 to 207.10 copies. The reduction was extremely significant (|t|=8.144, P=0.001, paired t-test) by 29.00±23.91 times on average. These results did not included the egg sample of the 10-year-old female silver carp because it was contaminated with blood.

### Mitogenome-wide landscape of heteroplasmy

**Fig. 1.**
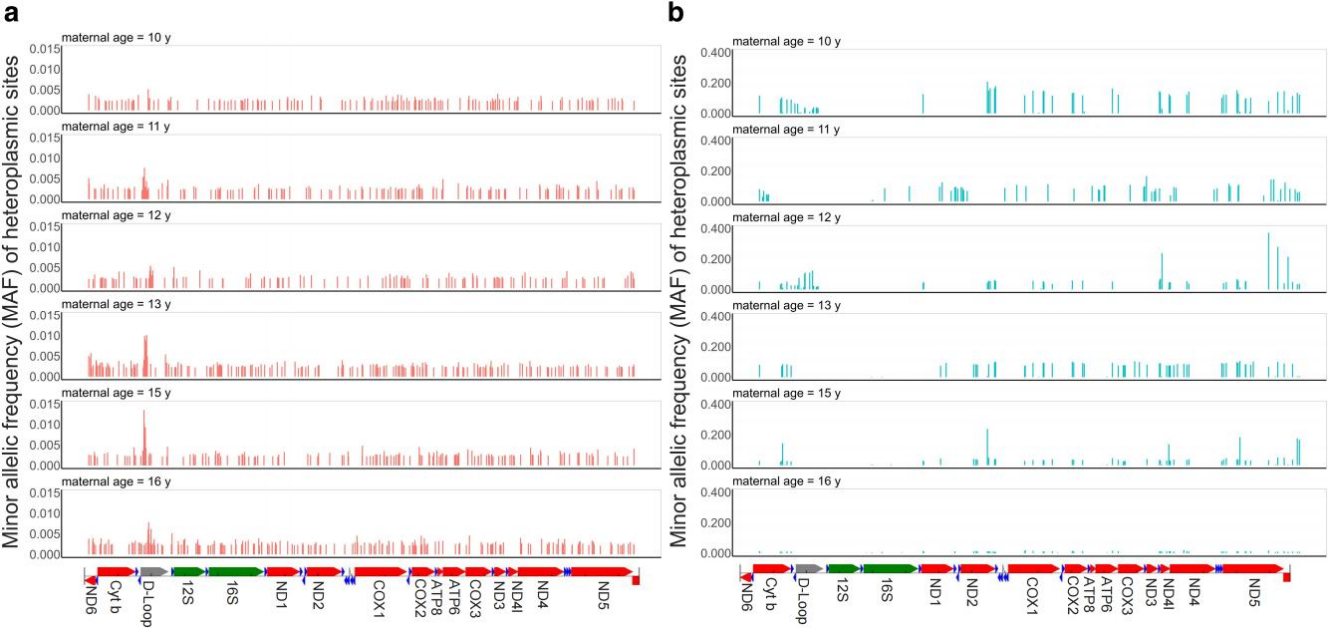
Landscape of HP sites and minor allelic frequency (MAF) across the mitogenomes of eggs. a) and hatchlings, b) of silver carp.

The reference mitogenome IntraRs were successfully assembled for all female silver carps, with the length between 16,614 bp and 16,616 bp. Varients were called and filtered using the IntraRs for both eggs and hatchlings are all SNPs. It showed SNPs or HP sites distributed across the whole mitogenome in both eggs and hatchlings, encompassing all functional regions (Fig. 1a, b). On average, the mitogenomes of eggs of each female carp contained 169.5 HP sites, while this number of hatchlings was 64.5. In eggs, the regions with the highest proportions of HP sites were *COX1* (11.9%), *rrnL* (10.7%) and *D-loop* (10.6%), while the regions in hatchlings were *ND5* (22.5%,), *ND3* (11.6%), and *ND2* (11.1%) (Table 2).

**Table 2.** Minor allelic frequency (MAF) and proportion of HP sites in different regions of silver carp mitogenome.

	Minor allelic frequency (%)		Proportion of HP sites (%)	
Region	Eggs	Hatchlings	Eggs	Hatchlings
D-loop	0.4	2.1	10.6	9.0
ATP6	0.3	6.4	3.0	6.5
COX1	0.3	6.2	11.9	8.5
COX2	0.3	5.7	5.7	2.1
ND2	0.3	7.7	3.1	11.1
ND3	0.3	6.9	2.7	11.6
ND4	0.3	6.5	7.6	8.0
ND5	0.3	6.8	9.2	22.5
rrnL	0.3	3.3	10.7	3.1

**Fig. 2.**
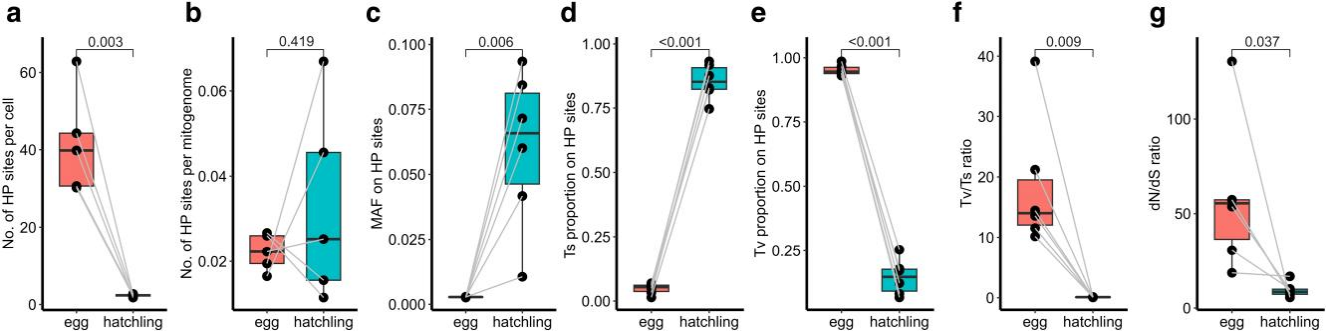
Trans-generation changes of mitogenome heteroplasmy. a) The number of HP sites per cell, b) The number of HP sites per mitogenome, c) Minor allelic frequency (MAF) on HP sites, d) Proportion of transition (Ts) in total substitutes on HP sites, e) Proportion of transversion (Tv) in total substitutes on HP sites, f) Tv/Ts ratio, and g) d*N*/d*S* ratio.

The average number of HP sites was 41.55±13.34 per egg, and 2.32±0.35 per cell in hatchling (|t|=6.599, P=0.003, paired t-test, Fig. 2a). The average number of HP site per mitogenome was 0.02±0.00 in eggs and 0.03±0.02 in hatchling, but the difference was not significant (|t|=0.900, P=0.419, paired t-test, Fig. 2b).

The minor allele frequency (MAF) of HP sites ranged from 0.21% to 1.3% with the mean of 0.28%±0.01% in eggs, and from 0.21% to 37.18% with the mean 6.03%±3.04% in hatchlings. The difference was significant (|t|=4.625, P=0.006, paired t-test, Fig. 2c). The greatest MAF was 0.4% in *D-loop* region of eggs and 7.7% in *ND2* region of hatchlings (Table 2).

For substitution types, transition (Ts) containing AT→GC/GC→AT accounted for 4.77%±2.07% of the total SNPs in eggs and 85.38%±6.94% in hatchlings. The difference was extremely significant (|t|=33.32, P<0.001, paired t-test, Fig. 2d). On the other hand, transversion (Tv), most of which were AT→TA, accounted for 95.23%±2.07% in eggs and only 4.62%±6.94% in hatchlings (|t|=33.36, P<0.001, paired t-test, Fig. 2e).

Furthermore, we tested the selection pressure based on Tv/Ts ratios for the whole mitogenomes and dN/dS ratios on mtCDS, respectively. The Tv/Ts ratio was 18.34±10.88 for eggs and 0.086±0.029 for hatchlings (|t|=4.117, P=0.009, paired t-test, Fig. 2f); dN/dS ratio was 58.03±38.98 for eggs and 9.44±3.93 for hatchlings (|t|=3.78, P=0.037, paired t-test, Fig. 2g). These results suggested that the mitogenomes were under stronger positive selection in eggs than in hatchlings.

### Genetic loads on mitogenomes

**Fig. 3.**
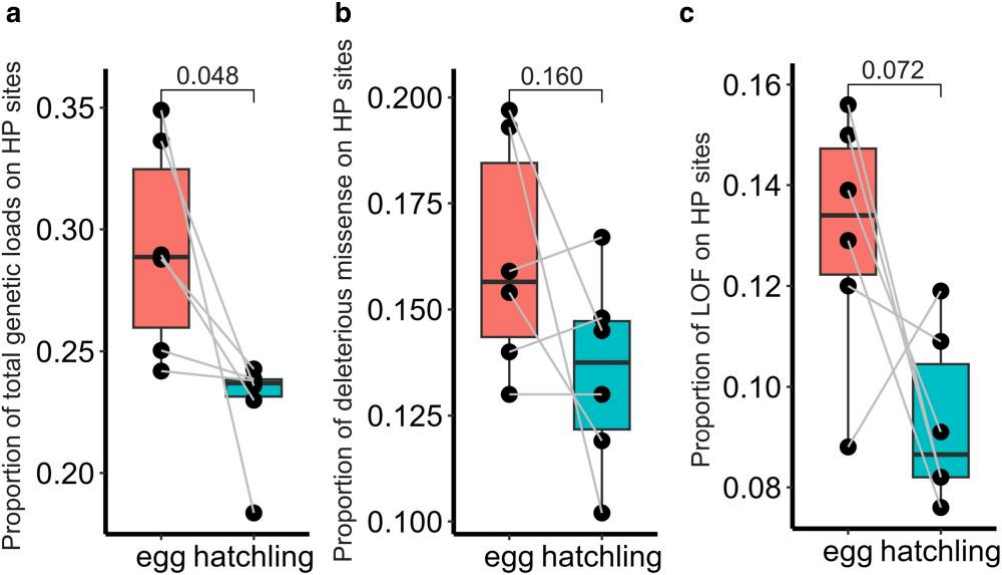
The proportion of genetic loads in total heteroplasmic substitutes on the mitogenomes of eggs and hatchlings. a) Total genetic loads including deleterious missense and loss of function (LOF) mutations, b) Deleterious missense mutations, and c) LOF mutations.

We tested genetic loads including deleterious missense mutation (Grantham score >150) and loss of function (LOF) mutation in CDS regions of mitogenome. The total genetic load was 0.293±0.044 in eggs and 0.228±0.022 in hatchlings, respectively (|t|=2.609, P=0.048, Fig. 3a). The deleterious missense mutation was 0.162±0.027 for eggs and 0.135±0.023 for hatchlings, respectively (|t|=1.657, P=0.160, Fig. 3b), while the LOF was 0.130±0.025 for eggs and 0.093±0.017 for hatchlings, respectively (|t|=2.275, P=0.072, Fig. 3c).

## Discussion

### Data quality

Nuclear mtDNA (NUMT) may provide fake signal of heteroplasmy (Xue *et al.* 2023). To avoid this, we detected and excluded NUMTs from the data of all samples by using blastn and Lastz methods. The blastn is the most commonly used tool that uses extant mtDNA sequences as probe. However, it better performs for relatively recent NUMTs (Mishmar *et al.* 2004). A total of only 3 short NUMTs were detected by this means. The Lastz is more sensitive to long NUMTs than blastn, but only one NUMT was detected (Table 1). It is hard to expect the chromosomal genome of silver carp contains only four NUMTs (Antunes and Ramos 2005). However, the heteroplasmic pattern in Fig. 1 showed high frequency of minor alleles in D-loop region of eggs but not in the same region of hatchlings. Given the heteroplasmy signals were significantly influenced by NUMTs, the two sorts of samples would be more similar. This suggests that the heteroplasmic signal we detected for all samples were trustable enough, even though the influence of NUMTs can be eliminated.

### Heteroplasmic landscape characteristics of mitogenome in the silver carp

The egg of fish experiences massive mitogenome replications during development and maturation that generates *de novo* substitutions (Otten, Stassen, *et al.* 2016). Our study reports the heteroplasmic landscape of mitogenome in egg and hatchling in the silver carp for the first time. Heteroplasmy happens across the whole mitogenome in both eggs and hatchlings (Fig. 1). For each HP site, substitution was detected only once with low frequency, suggesting heteroplasmy is dominated by recent *de novo* mutations. There was no differences in the number of HP site per mitogenome between the eggs (0.02±0.00) and hatchlings (0.03±0.2, P=0.419). This suggest that the heteroplasmic substitution occurs at constant rate so that the number on each mitogenome is stable despite of the cell type they belong to. Dominant substitutions were transversion with low frequency (mean 0.28%±0.01%) taking 95.23% of all Hp sites per egg (mean 41.55±13.34), while transition mutations with higher frequency (6.03%±3.04%, P=0.006) were dominant in hatchlings, taking 85.38% of all Hp sites per somatic cell (2.32±0.35, P=0.003). The region containing the highest number of HP sites was *COX1* for eggs and *ND5* for hatchlings, while the region with highest MAF was *D-loop* for eggs and *ND2* for hatchlings (Table 2). Such contrasting results reveals that different biological settings between eggs and somatic cells of hatchlings could shape the mitochondrial heteroplasmic landscape and make it compatible for the functional requirements (Li *et al.* 2015; Sanchez-Contreras *et al.* 2023).

### Quality control, selection, and trans-generation dynamics of mitogene pool

It’s known that different mitogenome variants have different impacts on cellular processes. For instance, mutations in noncoding region (*D-loop*) and RNA coding regions may alter the conformational stability of core elements required for replication and transcribed RNAs (Nicholls and Minczuk 2014), while those happening in protein-coding regions may lead to respiratory chain defects by producing excessive ROS and impairing OXPHOS system (Murphy 2009). However, malfunctioning mitochondria can be removed by selective mitophagy (Mizushima *et al.* 2008; Carelli *et al.* 2015; Knorre 2020) together with variants from the mitogene pool (Nunn and Goyal 2022) retaining variants with slight or no deleterious effects or favored variants with beneficial effects.

We detected strong positive selection in eggs (Fig. 2f, g) that results in retention of a large number of excessive Tv on the mitogenomes (Fig. 2e). This demonstrates that selection in eggs favors nonsynonymous substitutes that lead to changes of encoded RNAs and proteins. However, such substitutes contained a lot of deleterious and even LOF genetic loads, implying positive selection in eggs favors or is at least tolerant to the deleterious substitutes. This interesting phenomenon conforms with a scenario of the “survival of the slowest” hypothesis (De Grey 1997). The hypothesis proposes that intact mitochondria containing normal mitogenomes have usual ability to pump protons across the inner membrane. The protons can react with superoxide radicals to form perhydroxyl radicals, which can initiate membrane damage. While mitochondria with deleterious mitogenomes produce more superoxide radicals than intact ones and the ability to pump protons is partly impaired, so that the production of reactive perhydroxyl radicals is largely reduced. As consequence, the mitochondria with normal mitogenomes are more prone to degradation and faster membrane damage than the ones with deleterious mitogenomes (Kowald and Kirkwood 2000). The metabolism of oocyte is well-balanced and timed (Gu *et al.* 2015). The oocyte maturation involves increased energy supply and metabolic regulation (Nagahama and Yamashita 2008; Kang *et al.* 2021). If the “survival of the slowest” hypothesis is the case, it could be conjectured that the number of mitogenomes is more essential than the quality to meet the demand of oocyte metabolism. On one hand, the normal sequence is overwhelming at any nucleotide position in the mitogene pool as heteroplasmic variants are minor alleles (Table 2), so that the majority of mitochondrial products are normal. On the other hand, existence of a number of deleterious variants is conductive to reduce mitochondrial damage. By finely balancing between the number and quality of mitogenome, the oocyte is able to achieve an optimal state.

However, this raises a question immediately: can genetic loads in egg’s mitogene pool be transmitted to offspring and make deleterious impacts? The answer is definitely no, because otherwise all natural populations would decline and become extinct in later generations. Instead, the offspring mitogene pool should be well controlled for quality. First, mitochondria with severe deleterious mitogenomes can be removed from the egg through mitochondrial fragmentation (Lieber *et al.* 2019) by a unique programmed germline mitophagy during oogenesis (Palozzi *et al.* 2022). Second, mitochondria with high-inner membrane potential (likely due to nondeleterious variants) tend to be recruited preferentially into the mitochondrial cloud, transported along with germ plasm to the cortex of the vegetal pole and further become the initial mitogenome of the embryo after fertilization (Zhou *et al.* 2010; Bilinski *et al.* 2017; Tworzydlo *et al.* 2020). This process gives the opportunity to the healthy mitogenomes to pass through the bottleneck and initiate the offspring mitogene pool. Additionally, our study provides the third evidence that ontogenic process of hatchling mitogene pool is well controlled for quality. We observed that hatchlings had significantly less HP sites, greater variation of frequency of SNPs across the mitogenome (Fig. 1), lower genetic loads (Fig. 3), and weaker positive selection featured with excessive Ts (Fig. 2d, f, g) than eggs. Based on these results, we can reasonably speculate that the low-frequency Tv mutations in eggs can be discarded mildly, otherwise, it may have a direct impact on the survival and fitness of the offspring, Ts variants are then retained and progressively amplified within the cell with increasing frequency as the cell divides and mtDNA replicates.

**Fig. 4.**
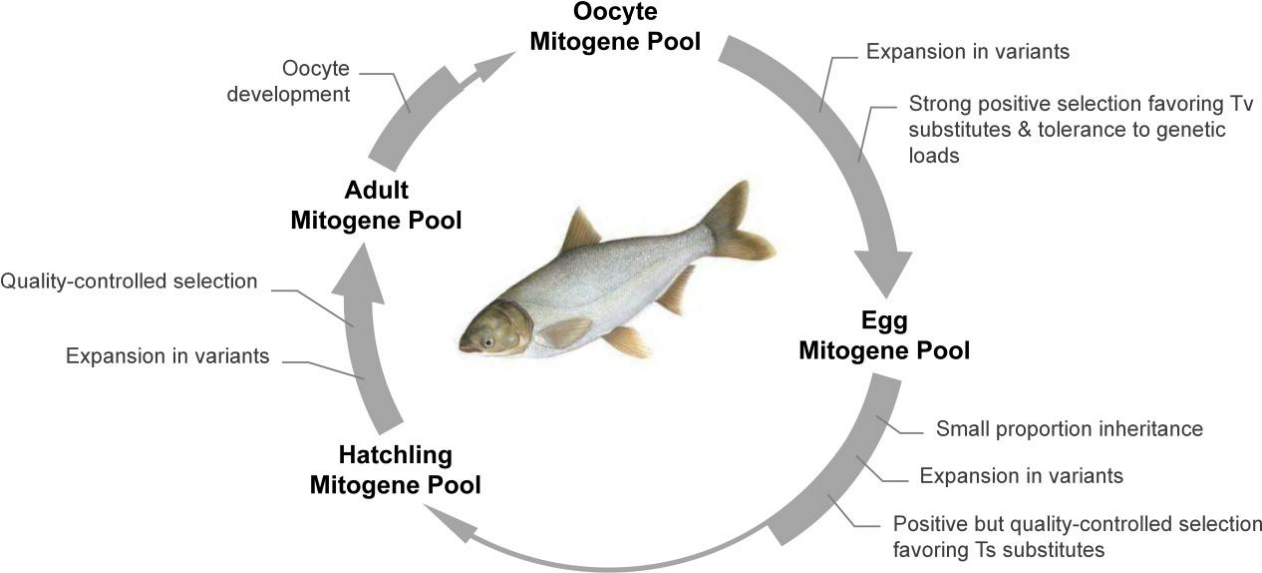
Trans-generation dynamics of mitogene pool in fish as represented by silver carp.

In general, the mitogene pool of eggs is a part of individual mitogene pool of the previous generation. It expands during oogenesis by massive replication, but replicons are selected in favor of variants with low membrane damage risks, which often carry genetic loads. A small proportion of qualified variants are able to pass the bottleneck as the initial mitogene pool of the next generation. Starting from the embryo, the mitogene pool develops under selection to ensure the quality of mitogenome population to fulfill the requirement of tissue function. Once again, the population involved in oogenesis develops into the egg mitogene pool and turning into the cycling (Fig. 4).

### Implications for future ecological studies and conservation

Traditionally, ecological studies and conservation practices center on genetic diversity of mitogenome at population or higher level. In such context, the data used are actually the consensus sequences of all mitogenomes in a sample, while the true mitogenomes in the sample are completely neglected. Our study shows the mitogene pool contains multiple variants of mitogenomes that raised from *de novo* replication errors during development. These variants are under selection, for instance, positively favoring genetic loads during egg development and negatively favoring healthy variants during ontogenesis of an individual. Although the effect of each variant is weak, the general effects would be strong enough to influence the functions of tissue, organ and further the individual holder. Therefore, taking the heteroplasmy into account in ecological study and conservation would provide additional information from the aspect of individual fitness and response to contemporary environment.

From our findings, several indices can be proposed to fulfill the requirements. The first is the functional volume of mitogene pool that can be defined as the total neutral and beneficial substitutes per cell. This index may measure the neutral and positive effects of total variants in a tissue controlled with copy number of mitogenomes in a cell. The second is the volume of load that can be defined as the total genetic loads per cell. It measures the potential deleterious effects of the mitogene pool. Selection strength is the third index to measure the strength the animal in response to contemporary environment. Comparisons of these indices among individuals or populations in the context of environment factors, geographic locations, climates, sexes, age classes, or life history stages, etc. would provide valuable information that are unaffordable from traditional approaches. Although they have not been empirically tested and validated in true populations, the characteristics and trans-generation dynamics of mitogene pool in this study provide strong implications for their validity and applicability.

In summary, this study quantitatively characterized the mitogenomic heteroplasmy including the landscape, genetic loads, selection strength in egg, and hatchling in the silver carp, and proposed the model of trans-generation dynamics to explain differential development mode of the mitogene pool between oocyte maturation and ontogenesis of offspring. It also sheds light on the value of mitogene pool for ecological studies and conservation practices.

## Supplementary Material

jkae101_Supplementary_Data

## Data Availability

The data that support the findings of this study have been deposited into CNGB Sequence Archive (CNSA) of China National GeneBankDataBase (CNGBdb) (https://db.cngb.org/) with accession number CNP0005128. Supplemental material available at G3 online.
