# Supplementary material for: Characterization and trans-generation dynamics of mitogene pool in the silver carp (Hypophthalmichthys molitrix)
Source: G3 (Bethesda). 2024 Jun 26;14(9):jkae101. doi: 10.1093/g3journal/jkae101 (PMC11491513; doi:10.1093/g3journal/jkae101)
Supplement: jkae101_Supplementary_Data [file jkae101_supplementary_data.docx]

**Supplementary figures**


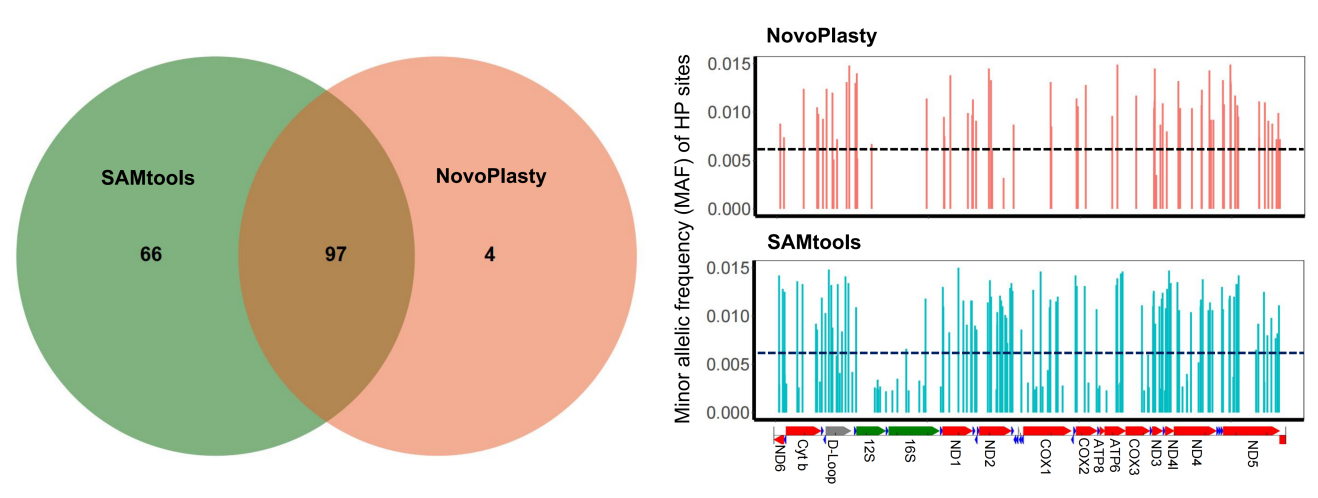


**Figure S1** Heteroplasmy (HP) sites and levels with SAMtools output compared with NovoPlasty. Venn diagram shows the HP sites detected jointly by both methods. The black dotted line represents the threshold of 0.6%(MAF) for NovoPlasty to detect HP levels.


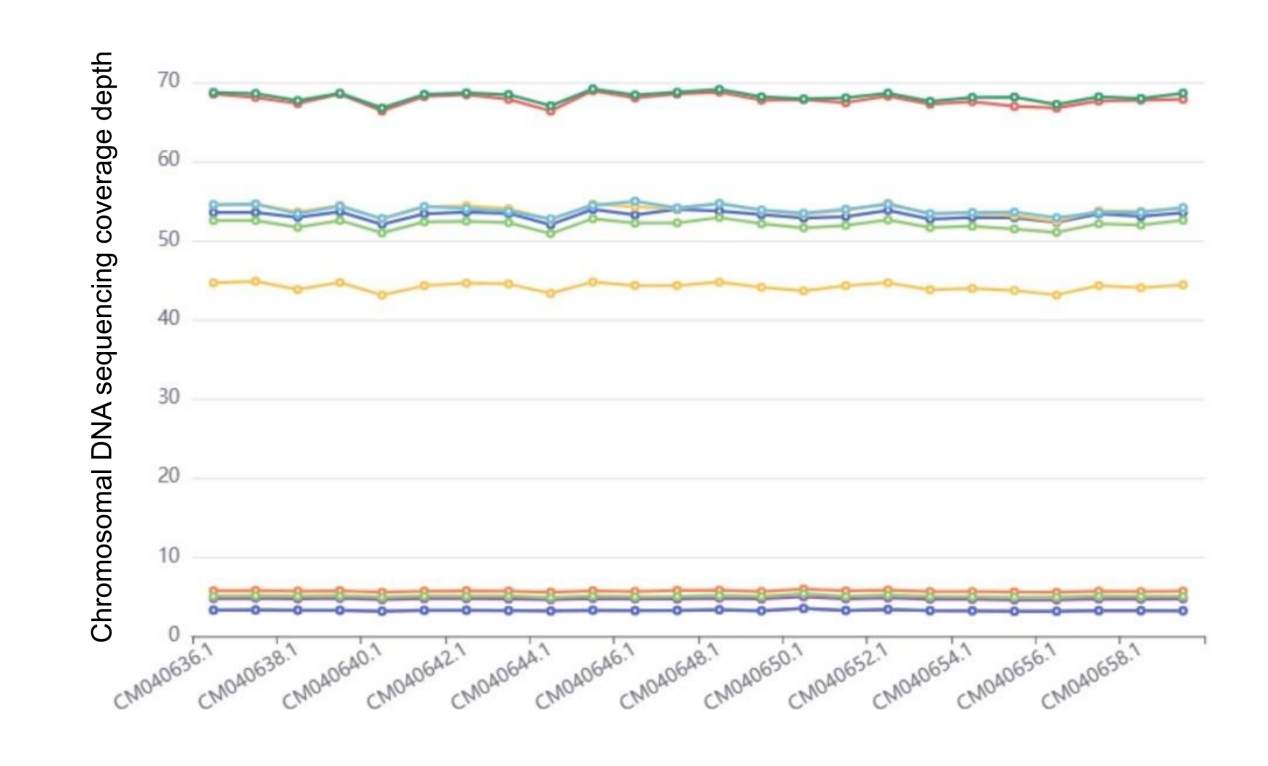


**Figure S2** Chromosomal DNA sequencing coverage depth among 24 Chromosomes
